# The need for an integrated approach for chronic disease research and care in Africa

**DOI:** 10.1017/gheg.2016.16

**Published:** 2016-11-29

**Authors:** A. L. Barr, E. H. Young, L. Smeeth, R. Newton, J. Seeley, K. Ripullone, T. R. Hird, J. R. M. Thornton, M. J. Nyirenda, S. Kapiga, C. A. Adebamowo, A. G. Amoah, N. Wareham, C. N. Rotimi, N. S. Levitt, K. Ramaiya, B. J. Hennig, J. C. Mbanya, S. Tollman, A. A. Motala, P. Kaleebu, M. S. Sandhu

**Affiliations:** 1 Department of Medicine, University of Cambridge, Cambridge, UK; 2 Wellcome Trust Sanger Institute, Genome Campus, Hinxton, UK; 3 Epidemiology and Population Health, London School of Hygiene & Tropical Medicine, London, UK; 4 Medical Research Council/Uganda Virus Research Institute (MRC/UVRI), Uganda Research Unit on AIDS, Entebbe, Uganda; 5 Global Health and Development, London School of Hygiene & Tropical Medicine, London, UK; 6 Malawi Epidemiology and Intervention Research Unit, Lilongwe, Malawi; 7 Mwanza Intervention Trials Unit, National Institute for Medical Research, Mwanza, Tanzania; 8 Department of Epidemiology and Public Health, Greenebaum Comprehensive Cancer Center and Institute of Human Virology, University of Maryland School of Medicine, Baltimore MD 21201 USA; 9 Institute of Human Virology, Nigeria; 10 Department of Medicine, University of Ghana Medical School, Korlebu, Ghana; 11 MRC Epidemiology Unit, University of Cambridge, Cambridge, UK; 12 Center for Research on Genomics and Global Health, National Human Genome Research Institute, National Institutes of Health, Bethesda, USA; 13 Division of Diabetic Medicine and Endocrinology, Department of Medicine, University of Cape Town, Cape Town, South Africa; 14 Shree Hindu Mandal Hospital, Dar es Salaam, Tanzania; 15 MRC Unit, The Gambia, Fajara, The Gambia; 16 MRC International Nutrition Group, London School of Hygiene & Tropical Medicine, London, UK; 17 Department of Internal Medicine and Specialties, Faculty of Medicine and Biomedical Sciences, University of Yaoundé I, Yaoundé, Cameroon; 18 MRC/Wits Rural Public Health and Health Transitions Research Unit, School of Public Health, Faculty of Health Sciences, University of the Witwatersrand, Johannesburg, South Africa; 19 INDEPTH Network, Accra, Ghana; 20 Department of Diabetes and Endocrinology, Nelson R Mandela School of Medicine, University of KwaZulu-Natal, Durban, South Africa

**Keywords:** Africa, chronic disease, health systems, implementation, integration, intervention, low and middle income countries, non-communicable disease, partnerships, research, surveillance, technology, infectious disease

## Abstract

With the changing distribution of infectious diseases, and an increase in the burden of non-communicable diseases, low- and middle-income countries, including those in Africa, will need to expand their health care capacities to effectively respond to these epidemiological transitions. The interrelated risk factors for chronic infectious and non-communicable diseases and the need for long-term disease management, argue for combined strategies to understand their underlying causes and to design strategies for effective prevention and long-term care. Through multidisciplinary research and implementation partnerships, we advocate an integrated approach for research and healthcare for chronic diseases in Africa.

## Burden of non-communicable diseases (NCDs) in Africa

Chronic NCDs such as diabetes, cardiovascular diseases (CVD), and cancers are emerging as leading causes of mortality and morbidity in Africa. In this region, with a population of around 1.1 billion, there were an estimated 28 million adults living with diabetes in 2015 [1, 2]. It is anticipated that in sub-Saharan Africa (SSA) alone, the number of people living with the disease will rise to 34.2 million by 2040 [2]. Similarly, in 2015, a projected 1.6 million deaths were attributable to CVD in Africa [3]. This figure is expected to rise by another 1 million by 2030 [3]. Cancer-related deaths are also anticipated to double to 1.2 million by 2030 [3]. Close to one-third of NCD-related deaths in low- and middle-income countries (LMICs) are premature and occur before the age of 60 [4]. Thus, NCDs already present a major health burden for the African continent and are expected to be the most common cause of death, exceeding the number of deaths from communicable, maternal, perinatal, and nutritional diseases combined, by 2030 [5].

## Risk factors for NCDs in Africa

Because of the diverse social, environmental, and biological settings within Africa, the distributions of known and other potentially novel risk factors, and their determinants, are likely to differ from those of European populations or those of African descent living outside Africa [6]. The higher incidence of certain cancers, such as liver, cervical and oesophageal, in Africa compared with high-income countries (HICs) may reflect underlying differences in their risk factors in these regions [7]. Population growth and the concomitant rise in life expectancy are likely to only partly explain the increase in NCDs. Many of the known risk factors for NCDs in HICs are the same for LMICs, including smoking, alcohol, diet, obesity, raised cholesterol and blood pressure [8, 9]. However, the distribution and relative contribution of these risk factors to the burden of NCDs in Africa are unclear. We also have only a limited understanding of the social, environmental and biological drivers of these risk factors within African populations. Rapid urbanisation and globalisation, and the associated trends towards unhealthy lifestyles, contribute to the burden of NCDs [10]. Small increases in urbanisation are associated with lower levels of physical activity and higher body mass index [11–13]. Globalisation has increased the availability of cheap, nutrient-poor, and energy-dense foods, which are likely to increase the risk of obesity and associated cardiometabolic risk factors [14]. Notably, the high burden of NCDs in rural areas, as well as urban centres, suggest there may be additional contributing or distinct factors [15].

## Impact of maternal and childhood health on NCDs in Africa

Maternal and neonatal health and risk of NCDs are interrelated. The prevalence of overweight and obesity in adult women in Africa has been rising; between 1980 and 2013, the burden of overweight and obesity in females increased by an average of 10% [16]. Maternal obesity increases the risk of developing gestational diabetes [17], and is associated with poor maternal and neonatal outcomes [18–20]. Women who develop diabetes and hypertension during pregnancy have an increased risk of type 2 diabetes (T2D), CVD, and metabolic syndromes [19, 21–23]. Children of obese or diabetic mothers also have a higher risk of metabolic disease in later life [24]. By contrast, under-nutrition *in utero* or in early life may also result in an increased risk of T2D and CVD in adulthood [25–28]. Adaptive responses to exposures *in utero* are thought to prepare the foetus for the postnatal environment [25]. Rapid changes in the nutritional environments, as seen in many LMICs, could lead to an increase in NCDs [25–27]. These maternal and developmental risk factors may have a social, environmental, or biological basis, and are intergenerational – highlighting the complexity of the epidemiological transitions across the African continent, and in other LMICs [28, 29, 30].

## Changing burden of infectious disease in Africa

Substantial progress has been made in reducing the burden of many types of infectious diseases, including those in early childhood. However, tuberculosis (TB), malaria, and HIV, as well as hepatitis B and C, remain endemic across the region. Africa has the highest burden of HIV in the world, with approximately 26 million prevalent cases and 1.3 million new infections recorded in 2014 [31]. TB and HIV co-infection is a growing issue in many LMICs [32]; as such, it is the most common cause of death for people with AIDS [33]. Anti-retroviral therapy (ART) coverage in Africa has rapidly increased over the past decade; 51% of known cases in SSA received treatment in 2012 [34]. Expanding use of ART has led to a notable decline in HIV-associated morbidity and mortality in Africa; HIV is rapidly becoming a chronic disease, requiring long-term treatment and management. This and the emergence of drug resistant strains of HIV, malaria, TB, and other pathogens pose a major challenge for the continent's infectious disease control and management programmes, which may also have implications for the burden of NCDs [35, 36].

## Interrelationship between non-communicable and infectious disease in Africa

The interrelated risk factors for infectious and non-communicable diseases are likely to have an important impact on the spectrum and distribution of chronic diseases in Africa, and other LMICs undergoing similar epidemiological transitions. The immune and metabolic systems are closely integrated, with each system's response dependent on the other for normal function [37]. Evolving from a common antecedent organ, they have shared and overlapping signalling pathways [37]. Chronic inflammation has been unequivocally linked to obesity, insulin resistance, T2D, and an increased risk of malignancy [37, 38]. Likewise, several infectious diseases and their treatments are associated with an increased risk of NCDs [39]. HIV and ART may increase the burden of cardiometabolic risk factors, including lipid and glucose abnormalities [40–43]. Hepatitis B and C infection may also increase the risk of developing T2D [44–46], as well as chronic liver disease and hepatocellular cancers, in addition to other oncogenic pathogens [47]. Similarly, insulin resistance and T2D may influence clinical outcomes in patients with hepatitis-associated liver disease and cancer [48]. Endemic infectious diseases may have also had an impact on selective adaptation and risk of NCDs – for example, renal function and African trypanosomiasis [49]. Thus, the body's own immune response, endemic and chronic infections, and their treatments, may play an important role in the development and progression of NCDs in Africa, although the underlying mechanisms are not well understood.

## The need for surveillance and prospective studies in Africa

Understanding the aetiology and determinants of NCDs in Africa is a fundamental step in developing strategies for disease prevention, management and control. Crucially, the impact of population and individual risk factors on disease susceptibility is largely unknown. Usually, chronic disease risk prediction models applied to African populations are based on regional comparisons of national indices, cross-sectional or case-control assessments, and the extrapolation of risk prediction algorithms developed in populations in Europe and elsewhere [50–52]. Furthermore, those studies conducted in African populations often use varying methods and definitions, or only assess a small subset of potential risk factors, limiting comparative analysis [53–55].

Whilst research institutions in Africa have clearly developed research frameworks for assessing the epidemiological and clinical burden of chronic disease across the region, there is a need to integrate and scale up such efforts [6, 40, 51, 56]. The INDEPTH Network of health and demographic surveillance systems is an example of a pragmatic model for examining disease burden across different settings [57–59]. Utilizing such established surveillance systems and analogous research initiatives to implement high quality and comparable large-scale population-based studies across the spectrum of chronic diseases and their risk factors will be crucial to understanding the aetiology and burden of chronic diseases in Africa. Likewise, implementation of standardised tools for the measurement of NCD risk factors in LMICs, such as those developed by the WHO, will enable comparability [60]. Importantly, establishing prospective studies in these settings will provide an invaluable framework to evaluate the utility of existing generic cut-off points or develop specific risk prediction algorithms for African populations, and provide the foundations for future aetiological and healthcare interventions.

## The need for research into the implementation of integrated health services and the management of chronic diseases in Africa

Aligning epidemiological and implementation research will provide the most effective strategy to identify pragmatic solutions for delivering chronic disease health care. The emerging double burden of chronic infectious and non-communicable disease imposes a substantial strain on limited healthcare resources and has implications for health policy and planning. In many African countries, where health systems are fragile, under-resourced or targeted primarily to infectious diseases, the capacity to effectively deal with the burden of chronic NCDs and accompanying co-morbidities is severely limited [61, 62]. These health system challenges will only become more apparent as the prevalence of NCDs increases.

Chronic infectious and non-communicable disease programmes in Africa have traditionally been distinct at all levels of healthcare provision. However, emerging evidence suggests that an integrated approach to the broad spectrum of chronic diseases may provide the most cost-effective mechanism for disease treatment and control due to the related underlying pathogenesis and strategies for management [63, 64]. Integrating NCD management with existing HIV/AIDS/TB, malaria, and maternal and child health programmes would utilise existing infrastructure, and allow for more rapid implementation of NCD health care [65]. Ideally these services would be placed within a strengthened and well-resourced primary health care system that can provide pro-active, patient-centric and long-term community-based care [66]. However, a more complete understanding of the broad range of risk factors, and their interrelation, will be critical in designing such integrated health care systems – and provide mechanisms for broader preventative strategies. In these contexts, it is vital to conduct public health implementation research to fully explore the most effective and economic strategies to deliver accessible and integrated health care for chronic diseases [67–70]. Universal implementation of existing low cost interventions for the diagnosis and management of NCDs and infectious diseases would be a pivotal first phase [66].

Likewise, it will be important to identify the most effective strategies for chronic disease management for the African context; reliable information on the efficacy of drug treatments for NCDs and infectious diseases, and their adverse reactions in African populations is limited [71, 72]. Harnessing and integrating existing health and pharmacovigilance systems could also facilitate drug efficacy trials and monitoring. Evaluation of current and novel chronic disease diagnostics, treatment and management strategies, including point of care testing and low-cost technology-based interventions within resource-limited settings, will be vital to implementing efficient mechanisms for integrated chronic disease research and care across the region, and in translating research findings into health care policy and services [73, 74].

## An integrated approach to chronic disease research and care in Africa

NCDs and infectious diseases should not be viewed as distinctive fields within global health research [75]. There is a critical need to combine research efforts across both acute and chronic infectious diseases and NCDs to better understand their interrelation and to develop more effective health systems to provide long-term management and care. Research and implementation partnerships will need to adopt innovative multidisciplinary research agendas that both strengthen and integrate existing infrastructure and advance implementation science. Combined, such structures could allow the formation of large-scale population health resources that would enable comprehensive studies into the diagnosis, prevention and management of chronic diseases, and their complex interactions, over diverse settings [76].
